# A diet high in fat and fructose adversely affects osseointegration of titanium implants in rats

**DOI:** 10.1002/cre2.255

**Published:** 2019-11-28

**Authors:** Shalinie King, Carina Baptiston Tanaka, Dean Ross, Jamie J. Kruzic, Itamar Levinger, Iven Klineberg, Tara C. Brennan‐Speranza

**Affiliations:** ^1^ Sydney Dental School, Faculty of Medicine and Health The University of Sydney Sydney New South Wales Australia; ^2^ School of Mechanical and Manufacturing Engineering UNSW Sydney Sydney New South Wales Australia; ^3^ Department of Physiology and Bosch Institute for Medical Research The University of Sydney Sydney New South Wales Australia; ^4^ Institute for Health and Sport (IHES) Victoria University Melbourne Victoria Australia; ^5^ Australian Institute for Musculoskeletal Science (AIMSS), Department of Medicine‐Western Health, Melbourne Medical School The University of Melbourne Melbourne Victoria Australia

**Keywords:** dental implant(s), histomorphometry, microcomputed tomography, osseointegration

## Abstract

**Objectives:**

Diet‐induced metabolic dysfunction such as type 2 diabetes mellitus increases the risk of implant failure in both dental and orthopaedic settings. We hypothesised that a diet high in fat and fructose would adversely affect peri‐implant bone structure and function including osseointegration.

**Materials and methods:**

Thirty female Sprague‐Dawley rats were divided into three groups (*n* = 10), control group (normal chow) and two intervention groups on a high‐fat (60%), high‐fructose (20%; HFHF) diet. Titanium implants were placed in the proximal tibial metaphysis in all groups either before commencing the diet (dHFHF group) or 6 weeks after commencing the diet (HFHF group) and observed for an 8‐week healing period. Fasting blood glucose levels (fBGLs) were measured weekly. Structural and functional features of the peri‐implant bone, including bone‐to‐implant contact (BIC), were analysed post euthanasia using microcomputed tomography, pull‐out tests, and dynamic histomorphometry.

**Results:**

The fBGLs were unchanged across all groups. Peri‐implant trabecular bone volume was reduced in the HFHF group compared with controls (*p* = .02). Percentage BIC was reduced in both HFHF group (25.42 ± 3.61) and dHFHF group (28.56 ± 4.07) compared with the control group (43.26 ± 3.58, *p* < .05) and reflected the lower pull‐out loads required in those groups. Osteoblast activity was reduced in both intervention groups compared with the control group (*p* < .05).

**Conclusion:**

The HFHF diet compromised osseointegration regardless of whether the implant was placed before or after the onset of the diet and, despite the absence of elevated fBGLs, confirming that changes in bone cell function affected both the initiation and maintenance of osseointegration independent of blood glucose levels.

## 1 INTRODUCTION

Titanium implants are an established treatment option for both dental and orthopaedic rehabilitation, particularly in an ageing population where demand is rapidly increasing (Srinivasan, Meyer, Mombelli, & Muller, 2017). Although 10‐year survival rates of 94.6% for dental implants (Moraschini, Poubel, Ferreira, & Barboza Edos, 2015), 95.6% for total hip replacements and 96.1% for total knee replacements have been reported (Bayliss et al., 2017), failures do occur. Metabolic dysfunction such as type 2 diabetes mellitus (T2DM) has been shown to be associated with reduced dental implant survival rates (Naujokat, Kunzendorf, & Wiltfang, 2016) and an increased risk for revision of total hip replacements (Pedersen, Mehnert, Johnsen, & Sorensen, 2010).

Normal bone remodelling processes are required for both initiation and long‐term maintenance of the bone‐to‐implant interface (Terheyden, Lang, Bierbaum, & Stadlinger, 2012). Recent evidence suggests that osseointegration is in fact a result of an immunologically driven foreign body reaction mounted by the body in response to the implant surface and that long‐term maintenance of this interface depends on equilibrium of this local inflammatory response, which attenuates over time (Albrektsson, Chrcanovic, Jacobsson, & Wennerberg, 2017; T. Albrektsson, Jemt, Molne, Tengvall, & Wennerberg, 2019). There is now growing evidence that patient‐related risk factors may impact on the maintenance of this interface (Maradit Kremers, Lewallen, van Wijnen, & Lewallen, 2016).

Diet, for example, can affect bone remodelling and, as such, the potential success of implants. Diets high in saturated fats have been shown to reduce trabecular bone microarchitecture in rodents (Lac, Cavalie, Ebal, & Michaux, 2008; Li et al., 2017; Macri et al., 2012; Yarrow et al., 2016), increase osteoclast activity, and expand bone marrow fat (Li et al., 2017; Yarrow et al., 2016). Diets high in fructose, which is metabolised in the liver into triglycerides and is associated with insulin resistance (Elliott, Keim, Stern, Teff, & Havel, 2002), have been shown to reduce the ex vivo osteogenic potential and increase the ex vivo adipogenic potential of marrow stromal cells (Felice, Gangoiti, Molinuevo, McCarthy, & Cortizo, 2014). A combination of high fat and high fructose has been shown to result in uncoupling of bone formation and resorption leading to reduced trabecular bone volumes (Wong, Chin, Suhaimi, Ahmad, & Ima‐Nirwana, 2018). Finally, a diet high in fat has been shown to adversely affect the initiation of osseointegration in both mini pigs and mice, respectively, by reducing the bone‐to‐implant contact and biomechanical properties of the interface (Coelho et al., 2018; Keuroghlian et al., 2015). The link between diet and bone metabolism may be provided by the fact that osteoblasts and adipocytes arise from a common mesenchymal stem cell within the bone marrow and that lineage selection can be affected by both local and systemic changes (Gregoire, Smas, & Sul, 1998). Importantly, diet‐associated negative effects on trabecular bone microarchitecture may be sex‐specific as the changes have been shown to be greater in male mice compared with female mice (Gautam et al., 2014). There are, however, no studies in female rodent models relating to diet‐induced changes in peri‐implant bone microarchitecture and osseointegration. Importantly, there are no studies on the effect of diet on the maintenance of osseointegration.

Changes in bone microarchitecture may be reflected in changes in function. Functional changes can be assessed by dynamic histomorphometry, which enables the quantitative assessment of bone formation over time and has been shown to be reduced in animals on a high‐fat diet (Tencerova et al., 2018). No studies have investigated diet‐induced changes to osteoblast cell function in peri‐implant bone.

Preclinical animal models enable analysis of modifiable factors that might affect osseointegration. As such, the aims of this study were to test the hypothesis that a diet high in fat and fructose in female rats adversely affects peri‐implant bone microarchitecture, osseointegration of a tibial titanium implant, and peri‐implant osteoblast cell function.

## 2 METHODS

### 2.1 Experimental design

Twenty female Sprague Dawley rats (aged 8 weeks) obtained from the Animal Resources Centre (Perth) were randomly divided into two groups (*n* = 10): control group (Control, AIN93G Rodent Diet) and a high‐fat, high‐fructose diet group (HFHF, SF02‐006 60% fat modification of AIN93G and 20% v/w fructose‐enriched water). Animals were maintained in individually ventilated cages, two rats per cage in a controlled temperature, humidity, and light environment with alternating 12/12‐hr light/dark cycles. Water and food were available ad libitum except when the animals were fasted for 6 hr prior to blood collections. Animals were acclimatised for a 2‐week period prior to the commencement of experimental procedures and monitored daily for the duration of the observation period. A sample size of five animals per group was determined in a power calculation based on bone‐to‐implant contact data presented in a T2DM rodent model (Wang et al., 2011), and this sample size was used for dynamic histomorphometry and pull‐out tests; however, all analyses of peri‐implant bone microarchitecture were performed on a sample size of 10 animals per group. The study protocol was approved by the University of Sydney Animal Ethics Committee, protocol Number: 2016/1009, and complies with the ARRIVE guidelines for reporting animal research (Kilkenny, Browne, Cuthill, Emerson, & Altman, 2010).

**Figure 1:**
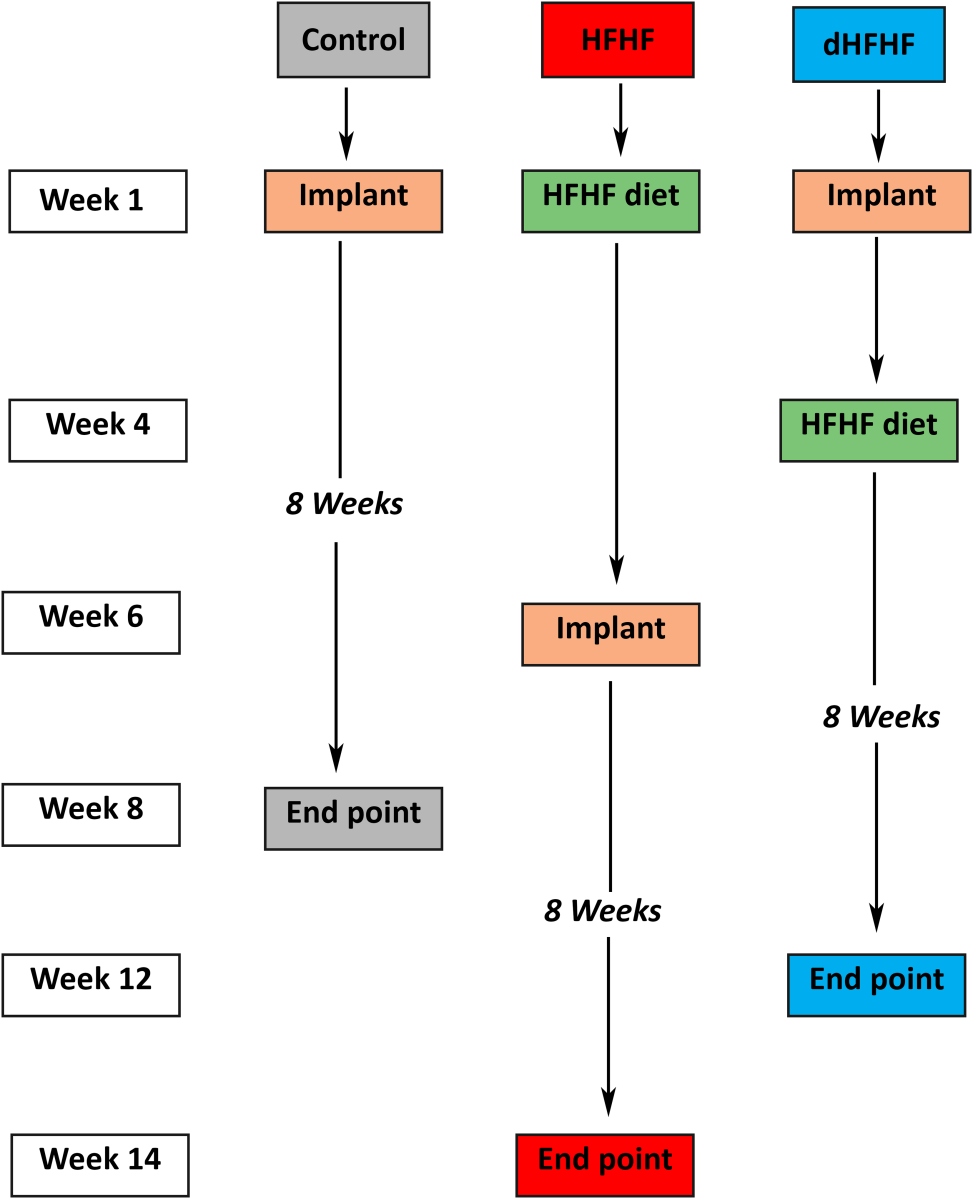
Timeline of the experiment design; both the control group and the HFHF group had implant healing times of 8 weeks, with the implants in the HFHF group being placed 6 weeks after the HFHF diet was introduced. The dHFHF group had an initial implant healing time of 4 weeks, the HFHF diet was introduced at Week 4, and the animals were maintained for a further 8‐week period. dHFHF, deferred HFHF; HFHF, high fat, high fructose

Implants were placed in the control group at Week 1 at the age of 10 weeks, and allowed to heal for an 8‐week period. The HFHF group commenced the HFHF diet at Week 1 at the age of 10 weeks, and the implants were placed at Week 6, when the animals were 16 weeks old. They were maintained on the HFHF diet and allowed to heal for a further 8‐week period (Figure 1).

To complement and extend this work, an additional group was included in order to determine whether placement of the implant in a metabolically healthy animal, with subsequent introduction of the HFHF diet 4 weeks post implant placement, would compromise osseointegration—this group was called the deferred HFHF group (dHFHF, *n* = 10), bringing the total number of animals used in the study to 30. In this group, the implant was placed at Week 1, at the age of 10 weeks, and the HFHF diet was commenced at Week 4 when the animals were 14 weeks old; they were then maintained for a further 8‐week period on the HFHF diet (Figure 1). The 3‐week healing period was chosen as initial bone remodelling has been shown to take place within 2 weeks in a canine model (Berglundh, Abrahamsson, Lang, & Lindhe, 2003).

For all groups, weekly fasting blood glucose levels (fBGLs) were measured following a 6‐hr fast using an ACCU Check Performa glucometer (Roche Diagnostic) and blood from the tail vein. Calcein (10 mg/kg, Sigma Aldrich) was provided intraperitoneally at Day 9 and Day 2 prior to sacrifice. At 8 weeks post implant placement for the control and HFHF groups and 8 weeks post commencement of the diet for the dHFHF group, the animals were anaesthetised with isoflurane, exsanguinated via cardiac puncture, and euthanised via decapitation. The right tibia was removed for microcomputed tomographic analysis, histomorphometry, and pull‐out testing.

### 2.2 Implant placement

**Figure 2:**
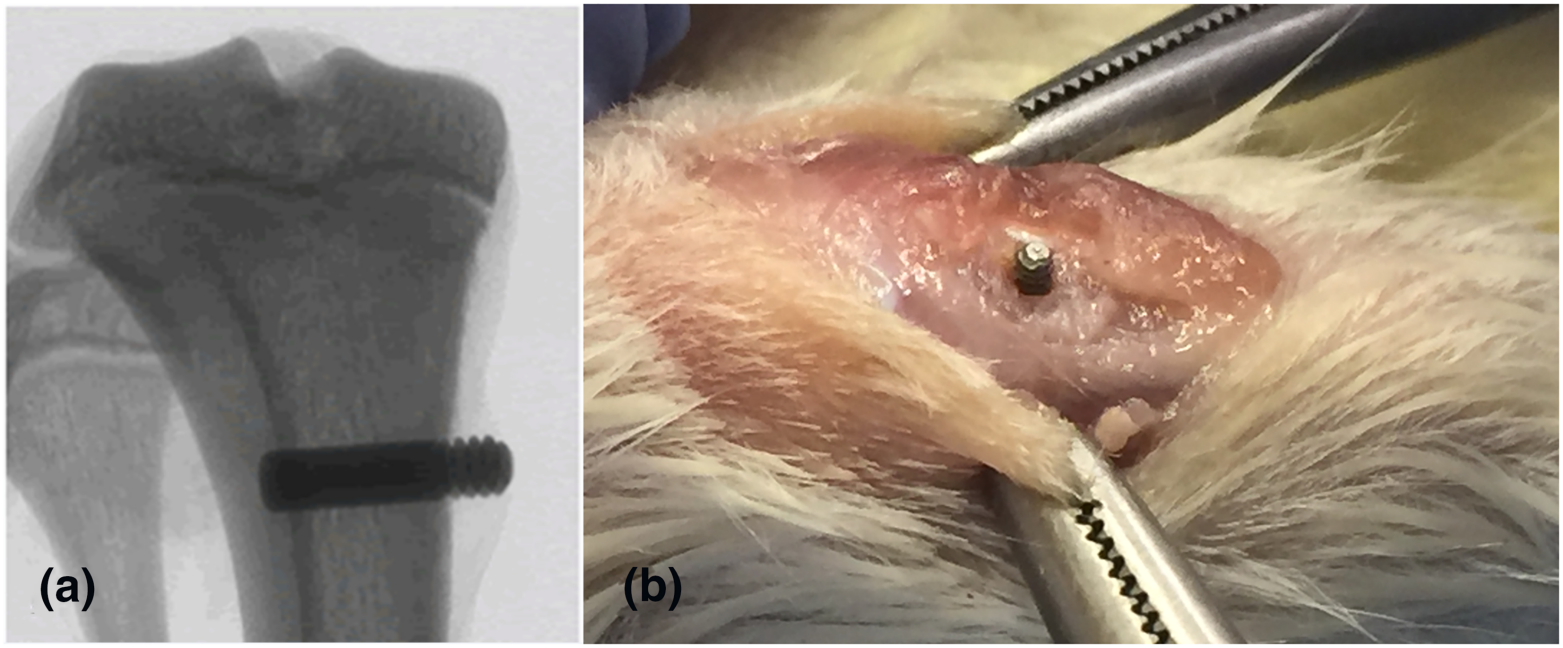
(a) Radiographic image of the implant positioned in the tibial metaphysis. (b) Photographic image of the implant positioned in the proximal tibia prior to achieving soft tissue closure

Animals were anaesthetised using a combination of 75‐mg/kg body weight ketamine (Ketamav 100, MAVLAB) and 10‐mg/kg body weight xylazine (Ilium Xylzil‐20, Ilium) administered intraperitoneally. The implant was inserted as previously described (Maïmoun et al., 2010; Figure 2). Soft tissue closure was achieved and sutured using 3‐0 resorbable sutures (Vicryl®). Postoperatively, the anaesthetic was reversed using Atipamazole (Ilium, Troy Laboratories), and, once the rats are awake, further postoperative analgesia (0.05‐ to 0.1‐mg/kg Buprenorphine [Temgesic®]) was provided immediately, followed by 1‐mg/kg of Meloxicam and Enrofloxacin 5 mg/kg (Baytril®50) for 4 and 5 days, respectively. One animal from each intervention group was lost due to anaesthetic complications.

### 2.3 Microcomputed tomography

Post sacrifice, the right tibia containing the implant was dissected out from each rat, excised, and fixed overnight in 4% paraformaldehyde and then transferred to 70% ethanol (ETOH). The specimens were wrapped in Parafilm to maintain hydration and scanned using the Bruker SkyScan. Each scan was performed with a 10‐μm voxel resolution, 0.14 rotation step, a projection time of approximately 590 ms, and 180° tomographic rotation. The source was set to 100 kVp at 100 μA, and an aluminium filter (0.5 mm) was used to minimise beam hardening artefacts. Scans were reconstructed in Nrecon (version 1.6.9.18) with all reconstruction parameters kept constant. Scans were then realigned using DataViewer (version 64 V1.5.2.4), and a volume of interest (VOI) was extracted from around the implant in the sagittal orientation. A 0.5‐mm × 1‐mm‐long region of trabecular bone was defined around the implant and analysed via CTan (version V1.16) with thresholds set for both bone and metal in order to segment the image. The 3D parameters relative bone volume (BV/TV), trabecular number (Tb.N), trabecular thickness (Tb.Th), and trabecular separation (Tb.Sp) were analysed within the VOI. The percentage of implant surface in direct contact with trabecular bone (bone to implant contact) within the VOI was measured following the Bruker method note (MN074) as a 2D measurement of the intersection surface.

### 2.4 Pull‐out tests

To conduct the pull‐out tests, five of the tibiae from each group were sectioned about 15 mm from the proximal end, partially embedded in epoxy resin and secured in a dynamic mechanical testing system (ADMET eXpert 5951, USA). A constant displacement rate of 1 mm/min was used to load the implant, with the load values recorded for the duration of the test. The pull‐out value was determined as the maximum load required to remove the implant.

### 2.5 Histomorphometry

The remaining five tibiae from each group were embedded in resin and prepared as previously described (Potres, Deshpande, Kloeppel, Voss, & Klineberg, 2016). Briefly, the sections containing the implant were dehydrated in ascending alcohol concentrations followed by incubation in 100% acetone and then a 1:1 mixture of acetone and infiltration media prior to embedding in Technovit 8100 Glycol Methacrylate at 4°C. A low‐speed, low‐deformation saw (Struers Accutom‐50) with a diamond wafering blade (12.7‐cm diameter, 0.4 mm thick—E015D) was used to section the resin blocks such that the implant was sectioned in half along the long axis. The sectioned block was then polished under water lubrication using silicon carbide paper (500, 800, 1,200, and 4,000 grits), and the polished surface of the block was glued to a microscope slide using clear epoxy resin and allowed to dry for 24 hr. The block with the glass slide attached was then returned to the low‐deformation saw, and a section of approximately 200 μm was obtained by sectioning the block 200 μm from the surface of the slide. The block attached to the slide was then ground further on the tegrapol‐25 to obtain a section thickness of between 60 and 80 μm.

**Figure 3:**
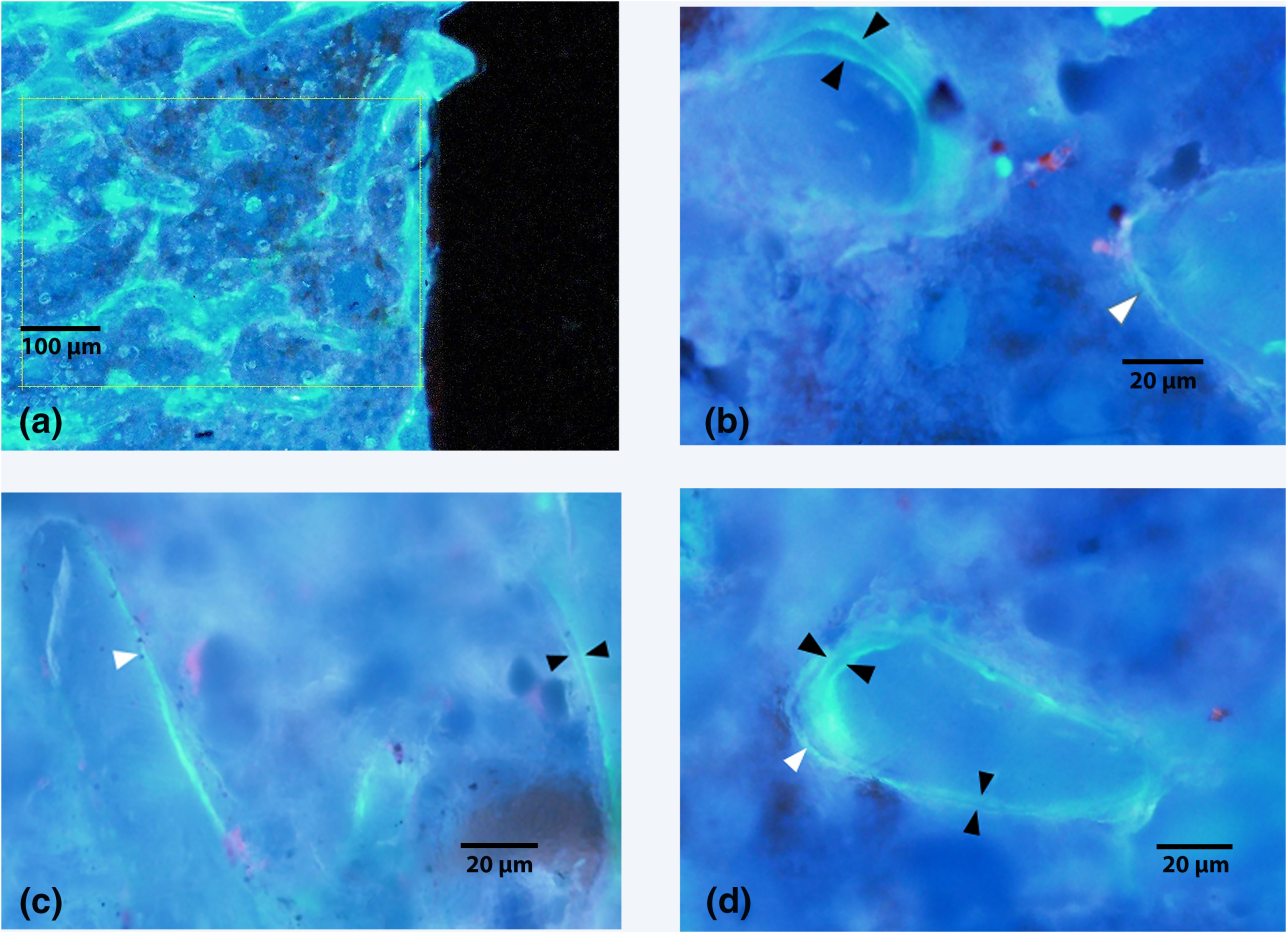
Representative micrographic photographs visualised under UV excitation through a Dapi filter, demonstrating the positioning of the first box (field of view) for dynamic histomorphometric measurements in an unstained, undecalcified section (4× magnification) (a). Higher magnification views of the peri‐implant region in the (b) control, (c) HFHF, and (d) dHFHF groups (20× magnification). The black arrow heads indicate areas of double labelling, and the white arrowheads indicate areas of single labelling. dHFHF, deferred HFHF; HFHF, high fat, high fructose

Histomorphometric analysis was performed on unstained, undecalcified 80‐ to 100‐μm thick sections at ×20 magnification in a series of three boxes (1,000 μm × 1,000 μm) positioned in trabecular bone on either side of the length of the implant beginning at the top end of the implant using the OsteoMeasure system (OsteoMeasure^TM^, Osteometrics Inc.; Parfitt et al., 1987; Figure 3). The dynamic parameters of mineral appositional rate (MAR), the mineralising surface per bone surface (MS/BS), and the bone formation rate (BFR/BS) were measured. Measurements were made by one blinded and calibrated examiner and 20% of specimens remeasured following a 2‐week interval with an intraclass correlation coefficient of.98.

### 2.6 Statistical analysis

Gaussian distribution of data was assessed using the Shapiro‐Wilk test, whereas data that were not normally distributed were log transformed. Differences between groups were analysed using a one‐way analysis of variance followed by Tukey post hoc multiple comparisons analysis in Graphpad Prism 7.0 (University Licence, San Diego, CA, USA). Repeated measures over time (only for fasting blood glucose) were analysed using a general linear mixed model repeated measures analysis followed by a post hoc multiple comparisons (Bonferroni) in SPSS (IBM SPSS Statistics 24, USA). The significance level was set at *p* < .05 for all analyses, and results are presented as Mean ± *SEM* or as box plots (median with interquartile ranges). The magnitude of change in the pull‐out tests was assessed using Cohen's effect size (ES), which was defined as small, 0.2; moderate. 0.5; and large, 0.8 or very large, 1.2 (Cohen, 1988).

## 3 RESULTS

### 3.1 Fasting blood glucose levels

**Figure 4:**
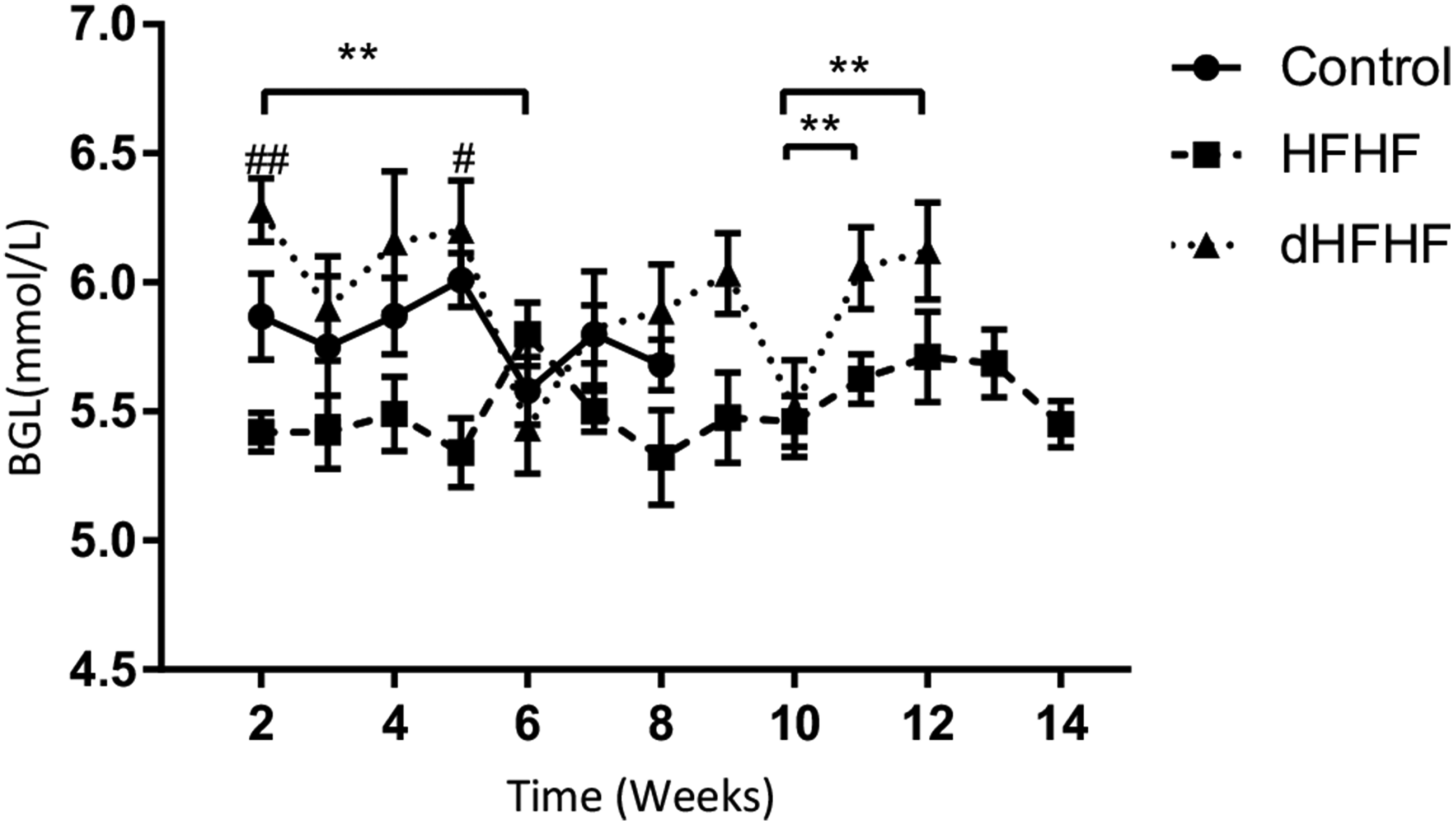
Fasting blood glucose levels (mmol/L): Week 2–Week 8 for the control (*n* = 10), Week 2–Week 14 for the HFHF (*n* = 9), and Week 2–Week 12 for the dHFHF (*n* = 9) groups. Results are presented as mean ± *SEM*. ^*^ *p* < .05 and ^**^ *p* < .01 for differences within groups over time; ^#^ *p* < .05 and ^##^ *p* < .01 for the HFHF compared with the dHFHF group as evaluated by a general linear mixed model repeated measures analysis followed by a post hoc multiple comparisons (Bonferroni test). dHFHF, deferred HFHF; HFHF, high fat, high fructose

To determine the effect of the HFHF diet on blood glucose levels, fBGLs were measured weekly; however, the Week 1 data were excluded due to a procedural error in blood collection for the control group in that week—the fBGL was measured after induction of anaesthesia (the anaesthesia was for collection of plasma for data not reported in this study), and this resulted in a transient hyperglycaemia. This error was not repeated in subsequent measurements, and the fBGLs were measured prior to induction of anaesthesia. At Week 2, there were no significant differences in fBGLs between the control group (6.9 ± 0.48 mmol/L) and the HFHF group (5.7 ± 0.08 mmol/L) or the dHFHF group (6.3 ± 0.12 mmol/L). Neither were there any differences at the final time point between the control group (5.35 ± 0.17 mmol/L), the HFHF group (5.5 ± 0.18 mmol/L), and the dHFHF group (5.9 ± 0.18 mmol/L). The dHFHF group was the only group to show significant changes in fBGLs during the study period (Figure 4).

### 3.2 Peri‐implant bone microarchitecture

To evaluate diet‐induced changes in bone microarchitecture, peri‐implant samples were analysed 8 weeks following implant placement in the control and HFHF groups and 12 weeks following implant placement in the dHFHF group. Both the BV/TV and Tb. N in the peri‐implant region were significantly higher in the control group compared with the HFHF group but not the dHFHF group. Additionally, the Tb. Sp for the control group was significantly lower than that of the HFHF group but not the dHFHF group. Finally, the Tb. Th for the control group was not significantly different to either the HFHF or the dHFHF group (Table 1).

**Table 1:** Trabecular bone microarchitecture parameters in the peri‐implant bone

Parameters	Control	HFHF	dHFHF
Relative bone volume (BV/TV; %)	22.12 ± 8.00	11.96 ± 7.41*	14.97 ± 7.05
Trabecular thickness (Tb.Th; mm)	0.09 ± 0.02	0.07 ± 0.02	0.08 ± 0.02
Trabecular separation (Tb.Sp; mm)	0.165 ± 0.01	0.184 ± 0.01**	0.175 ± 0.01
Trabecular number (Tb.N; 1/mm)	2.5 ± 0.54	1.52 ± 0.55*	1.85 ± 0.68

*Note.* Control (*n* = 10), HFHF (*n* = 9), and dHFHF (*n* = 9). Data are expressed as Mean ± *SD.*

Abbreviations: dHFHF, deferred HFHF; HFHF, high fat, high fructose.

* *p* < .05 HFHF compared with the control group as evaluated by a one‐way analysis of variance with post hoc multiple comparisons (Tukey test).

** *p* < .001 HFHF compared with the control group as evaluated by a one‐way analysis of variance with post hoc multiple comparisons (Tukey test).

### 3.3 Osseointegration

**Figure 5:**
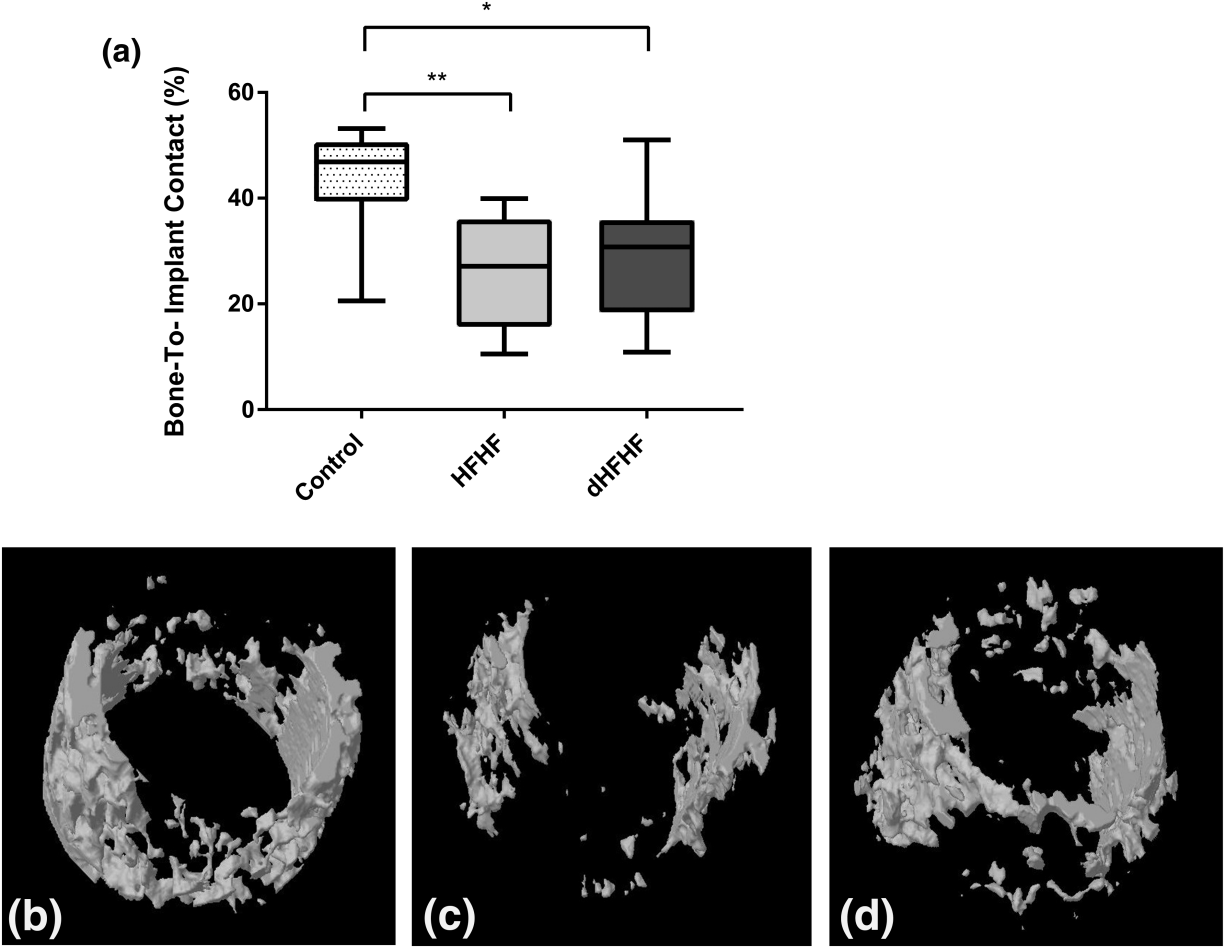
Osseointegration is compromised in the HFHF and the dHFHF groups (*p* = .006 and *p* = .03, respectively) 8 weeks following implant placement for the control and HFHF groups and 12 weeks following implant placement for the dHFHF group. (a) Percent bone‐to‐implant contact. Results are expressed as box‐and‐whisker plots with the median, minimum, and maximum values; control (*n* = 10), HFHF (*n* = 9), and dHFHF (*n* = 9). ^*^ *p* < .05 and ^**^ *p* < .01 compared with the control group as evaluated by a one‐way analysis of variance with post hoc multiple comparisons (Tukey test). (b–d) Reconstructed microcomputed tomography images of (b) control, (c) HFHF, and (d) dHFHF. dHFHF, deferred HFHF; HFHF, high fat, high fructose

To investigate the effect of a diet high in fat and fructose on osseointegration, the percentage of peri‐implant bone in contact with the implant was determined and found to be significantly reduced in both the HFHF (25.42 ± 3.61%, *p* = .006) and dHFHF groups (28.56 ± 4.07%, *p* = .03) compared with the control group (43.26 ± 3.58%). The reduction in bone‐to‐implant contact was more marked in the HFHF group, which started the diet prior to implant placement (Figure 5). The bone‐to‐implant contact in the dHFHF group was significantly lower than the control group despite the implant being in place for a longer period (12 weeks compared with 8 weeks as the implant in the HFHF group was placed 4 weeks prior to the commencement of the diet).

### 3.4 Pull‐out tests

**Figure 6:**
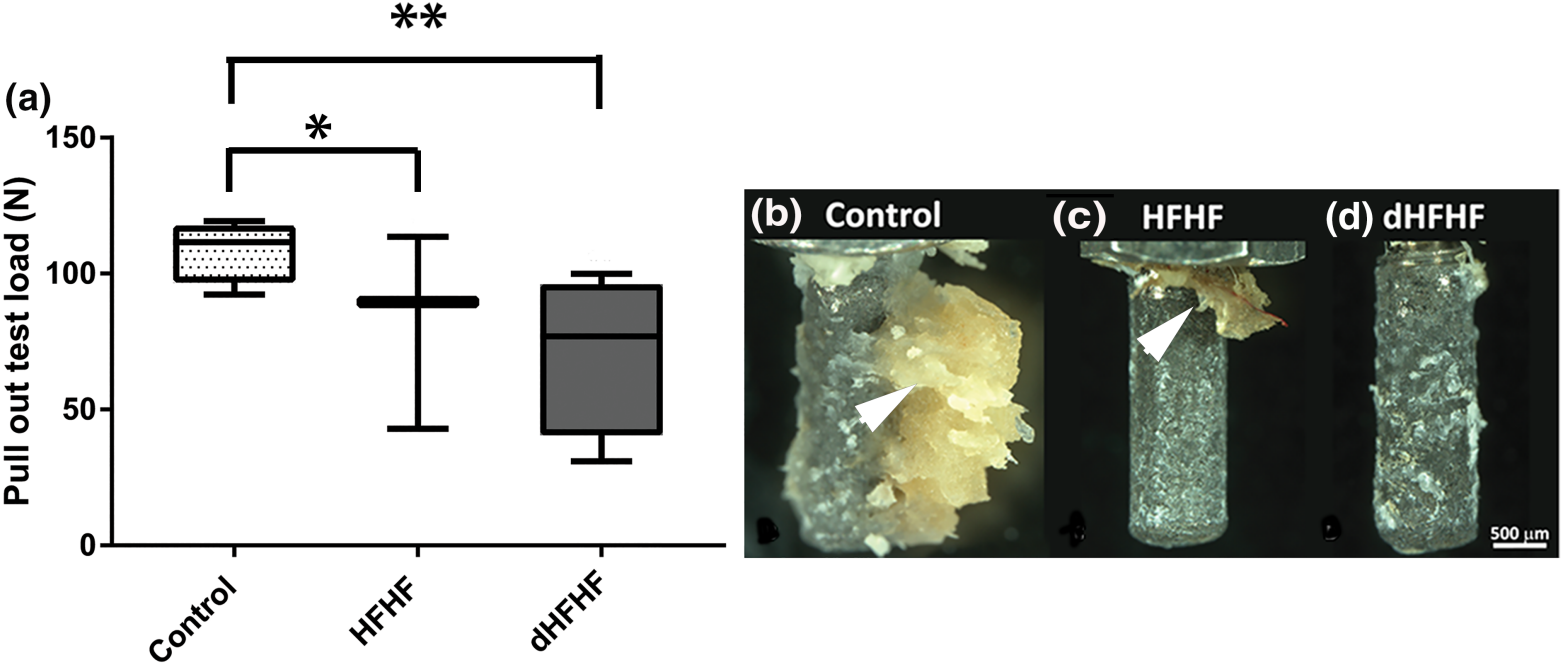
The load required to break the bone‐to‐implant interface is higher in the control group compared with both intervention groups although not statistically significant. (a) Load (N) required to remove the implant from bone. Results are expressed as box‐and‐whisker plots with the median, minimum, and maximum values; ^*^large effect size and ^**^very large effect size compared with the control group. Control (*n* = 4), HFHF (*n* = 3), and dHFHF (*n* = 4). Photographic images of the implant after removal from the tibia: the black arrowheads indicate bone tissue attached to the implant surface—with an increased amount of bone attached to the control implant (b) as compared with implants from the HFHF group (c) and dHFHF group (d). dHFHF, deferred HFHF; HFHF, high fat, high fructose

A reduction in bone‐to‐implant contact would be expected to reduce the biomechanical strength of the interface; to test this hypothesis, the load required to remove the implant was measured and found to be lower in both the HFHF (82.03 ± 20.71 N, *p* = .42) and dHFHF (71.21 ± 14.80 N, *p* = .17) compared with the control group (108.64 ± 5.8 N; Figure 5a). Some implants were partially cut during mechanical gripping and fractured upon loading prior to producing pull‐out force data. This reduced the sample size and thus the statistical power, making accurate differences between groups difficult to identify. Therefore, Cohen's ES was used in order to investigate the magnitude of the difference between the group means to provide an indication of the strength of the effect of diet on osseointegration. Samples sizes in Figure 3 are as follows: control, *n* = 4; HFHF, *n* = 3; and dHFHF, *n* = 4. Cohen's ES function revealed a large ES (1.03) for the HFHF and a very large ES (1.72) for the dHFHF compared with the control group. Implants failing above 100 N in all groups showed significant bone adhesion following pull‐out as illustrated by the control sample in Figure 6b, whereas those failing below 100 N in all groups showed very poor adhesion of bone to the implant surface following pull‐out as demonstrated by the HFHF and dHFHF samples in Figure 6c,d.

### 3.5 Dynamic histomorphometry

To determine the cellular mechanisms of reduced trabecular bone parameters and reduced osseointegration, dynamic histomorphometric parameters of bone turnover in the peri‐implant region were analysed. The MAR and BFR/BS were both significantly reduced in the dHFHF group (*p* = .02 and *p* = .01, respectively) compared with the control group. The MAR was also significantly lower in the HFHF group (*p* = .04) compared with the control group. The MS/BS was not significantly different in either the HFHF or dHFHF group compared with the control group (Table 2).

**Table 2:** Dynamic histomorphometric parameters in the peri‐implant bone

Parameters	Control	HFHF	dHFHF
Mineral appositional rate (μm/d)	16.76 ± 10.39	5.32 ± 3.15*	4.01 ± 3.00†
Bone formation rate/bone surface (μm^3^/μm^2^/d)	55.22 ± 27.73	29.02 ± 18.95	12.87 ± 8.49†
Mineralising surface/bone surface (%)	344.17 ± 103.66	565.55 ± 143.22	453.19 ± 283.32

*Note.* Control (*n* = 5), HFHF (*n* = 5), and dHFHF (*n* = 5). Data are expressed as Mean ± *SD.*

Abbreviations: dHFHF, deferred HFHF; HFHF, high fat, high fructose.

* *p* < .05 HFHF compared with the control group.

† *p* < .05 dHFHF compared with the control group as evaluated by a one‐way analysis of variance with post hoc multiple comparisons (Tukey test).

## 4 DISCUSSION

In summary, we report that a diet high in fat and fructose did not significantly alter fBGLs in female rats. However, this diet did result in compromised peri‐implant trabecular bone microarchitecture, compromised bone‐to‐implant contact, and was associated with a reduction in the biomechanical properties of the interface. Furthermore, this diet was associated with reduced osteoblast function in the peri‐implant region.

The effect of a diet high in fat and sugar on fBGLs is not clear. Although diets high in fructose or fat and fructose (HFHF) have been reported to increase fBGLs in male rats at 3 and 8 weeks (Dupas et al., 2017; Huang, Chiang, Yao, & Chiang, 2004; Wong et al., 2018), other studies have reported that fBGLs in male rats were unaltered 8 weeks following high‐fructose feeding (Sanchez‐Lozada et al., 2007) and 16 weeks following high‐fat/high‐sugar feeding (Gerbaix et al., 2012). Similarly, we did not observe a significant change in fBGLs in female rats on an HFHF diet. However, despite the absence of a significant effect on fBGLs, we did observe changes in bone structure and function. This suggests that the changes in bone tissue may be independent of changes to circulating blood glucose levels. Similarly, a recent study in minipigs on a diet high in fat has reported compromised bone‐to‐implant contact regardless of the presence of hyperglycaemia (Coelho et al., 2018).

This is the first study to report diet‐induced changes in peri‐implant trabecular bone microarchitecture. Previous studies in rodent models have reported that diets high in fat compromise tibial and femoral trabecular bone microarchitecture (Lac et al., 2008; Macri et al., 2012; Yarrow et al., 2016). In this study, 8 weeks following implant placement, compromised trabecular bone microarchitecture was noted in the HFHF group compared with the control group. The 8‐week healing period corresponded to approximately 5 years in human terms (Andreollo, Santos, Araújo, & Lopes, 2012). Trabecular bone microarchitecture in the dHFHF group was not compromised following 8 weeks on the HFHF diet. Importantly, the implant in the dHFHF group was placed 4 weeks prior to the commencement of the HFHF diet, resulting in a total of 12 weeks of healing compared with 8 weeks of healing in the HFHF group and the control group. This may have resulted in improved peri‐implant microarchitecture in the dHFHF group.

This study is the first to report that bone‐to‐implant contact is compromised even when the HFHF diet is commenced after initial implant integration has occurred. Compromised bone‐to‐implant contact was noted in both the HFHF and dHFHF groups despite the increased healing time in the dHFHF group. A similar high‐fat‐diet‐induced reduction in bone‐to‐implant contact has been reported in female minipigs and male mice (Coelho et al., 2018; Keuroghlian et al., 2015). However, in both these studies, the high‐fat diet was commenced prior to implant placement. Trabecular bone outcomes have been reported to be worse in male compared with female rats (Gautam et al., 2014), and this study supports this finding, reporting a reduction of less than 20% in bone‐to‐implant contact compared with over 20% in male mice (Keuroghlian et al., 2015). The data from the pull‐out tests suggest that the biomechanical properties of the interface may be compromised in both the intervention groups, and the relatively less bone tissue adhered to the extracted implant (Figure 6) noted in these groups supports this finding.

Healing of the osteotomy occurs through a process of distance osteogenesis and contact osteogenesis. In the former case, osteoblasts migrate from within the native bone to the surface of the osteotomy and start forming new bone along the walls of the osteotomy. In the latter case, osteoprogenitor cells colonise the implant surface, differentiate to form osteoblasts, and form new bone along the surface of the implant (Davies, 1998). It is possible that the mechanism for distance osteogenesis is less compromised by the HFHF diet as bone can be formed by osteoblasts already present within the native bone microenvironment and that the reduced bone‐to‐implant contact in both intervention groups may reflect compromised osteoblast differentiation and bone formation that is required for contact osteogenesis to occur. Indeed, a high‐fat diet has been shown to reduce the osteogenic potential of bone marrow (Felice et al., 2014).

This is the first study to report on diet‐induced changes in peri‐implant trabecular bone osteoblast function. The MAR was reduced in both intervention groups, reflecting the reduced rate of matrix production by osteoblasts. A similar high‐fat‐diet‐induced reduction in tibial and vertebral MAR and BFR has been reported in male mice (Tencerova et al., 2018). The BFR provides a measure of both the volume of matrix production (MAR) and the area of bone surface covered by active osteoblasts (MS/BS) and was reduced in the dHFHF group. These findings suggest that a diet high in fat and fructose compromises osseointegration by adversely affecting peri‐implant osteoblast function. The high fat content of the diet could result in an increase in plasma free fatty acids, which have been shown to stimulate the production of proinflammatory cytokines by osteoblasts, in turn leading to bone loss (Frommer et al., 2017).

A limitation of this study was that the effect of hyperglycaemia on peri‐implant bone could not be determined because fBGLs did not increase over time. The key aims of this study were to assess the effect of diet‐induced metabolic dysfunction on both the initiation and maintenance of osseointegration; this meant that the groups had to be of different ages at the end of the study period. Differences in ages were due to the need to test implant healing before and after the commencement of the diet with a standardised implant healing time of 8 weeks for each group. Ideally, the use of two control groups to match each of the intervention groups would have been useful but would have significantly increased the number of animals and was not possible due to constraints imposed by the Animal Ethics Committee. Although bone tissue function can be affected by age, in rodents, both trabecular and cortical bone has been shown to be stable between the ages of 12 and 24 weeks (Barbier et al., 1999). Lastly, the pull‐out tests resulted in damage to a number of implants, compromising the samples and making the final sample size too small for meaningful statistical analyses.

## 5 CONCLUSIONS

In conclusion, we have shown that an HFHF diet in female rats significantly compromises implant osseointegration despite the absence of metabolic changes to fBGLs and regardless of whether the implant is placed either before or after the onset of the diet. Changes in bone microarchitecture are supported by changes in cellular function over time. These changes indicate an underlying alteration in the regulation of osteoblast function and are an important step in understanding the mechanism behind the influence of diet on osseointegration. Diets high in fat and fructose could compromise the bone‐to‐implant interface and contribute to the development of late implant failures in patients with underlying systemic conditions such as T2DM. As such, dietary interventions could be considered in high‐risk patients requiring implants and in those with previously placed implants.

## CONFLICT OF INTEREST

The authors declare no potential conflicts of interest with respect to the authorship and/or publication of this article.

## AUTHOR CONTRIBUTIONS

Study design: S. K., I. K., and T. B. S. Study conduct: S. K., D. R., C. T., J. K., and T. B. S. Data interpretation: S. K., J. K., I. L., and T. B. S. Drafting manuscript: S. K. All authors revised the manuscript, gave final approval, and agreed to be accountable for all aspects of the work.
